# Dental Scientific Research

**Published:** 1920-09

**Authors:** 


### DENTAL SCIENTIFIC RESEARCH

It has been the clearly defined policy of the *Dental Cosmos* to spread the gospel of a scientific basis for all dental activities and a recognition of the essential importance of the scientific attitude of mind upon the part of the dental profession individually and collectively toward the problems comprised within the dental professional field.

Thirty years ago a plea for the scientific method in dentistry received scant consideration by the majority of the dental profession. The demand was for the so-called ''practical,'' the thing that could be done by the hands and was measurable in terms of direct pecuniary advantage. Any abstract discussion of the causes behind phenomena was ''theory,'' which in the popular conception was mere speculative guessing and unworthy of serious consideration.

The terms ''science,'' ''scientific'' and ''theory'' have gradually taken their normal places in the dental professional mind through the development of a correct understanding of their meaning; so that today they are not looked at askance or with suspicion, but are accepted as the only true basis for dental progress as well as of progress in all other human activities.

Every department of dentistry is being scrutinized and revised in the light of scientific standards and by scientific methods of investigation. Prosthetic dentistry is conforming to the laws of physics and chemistry and the engineering problems which it presents are being solved in harmony with the laws of mechanics. So likewise in orthodontia the same scientific considerations apply even in relation to the less evident biologic phenomena involved in the tissue changes incident to tooth movement; for it has been said by an eminent physiologist that even biologic phenomena, including the mutations of protoplasmic activity, must finally be expressed in terms of chemistry and physics.

Roentgenology in its relation to dental diagnosis has made a profound impression upon standards and methods of dental practice. Upon the evidence, more accurately the testimony, of the radiograph teeth are condemned to extraction, or saved, in accordance with the skill and understanding of the interpreter of the radiographic record, in spite of the fact that the physical phenomena, by which the X-ray record is made possible, are an impenetrable mystery to the majority of those who apparently feel themselves qualified to make an expert reading of the record and reach a diagnosis therefrom.

Those who have the most precise knowledge of roentgenology agree as to its present limitations as a diagnostic means and are impressed with the belief that, notwithstanding its present value, the application and possibility of the X-ray are capable of immense development in scientific precision with a greatly increased accuracy of interpretation.

In operative dentistry and in dental therapeutics the field is limitless for scientific investigation and standardization, for in dealing with the life and health of the dental tissues the empirical method is too heavily weighted with danger to human life and health to be longer tolerated when scientific study and research offers the best as indeed the only way to intelligent, rational and successful therapeutics.

The scientific method no longer needs supporting argument, for it has justified itself by its results, but what does appear to be required is intelligent direction so that economy of time and effort may be secured in the attainment of practical results. The scientific research activities in dentistry may be classified as:

1. Research as an individual enterprise.
2. Research promoted and fostered by dental organization.
3. Research in the industrial activities related to dentistry.

The first group will always comprise those workers who are seekers after the truth for the truth's sake and whose major compensation is the gratification of the creative instinct in the workers in pure science to whom the revelation of the truth is its own reward, and to whom pecuniary compensation is an anomaly except in so far as it is an aid to the attainment of the main objective. These are the inspired ones who are the dreamers of dreams, whose visions may or may not be immediately convertible into terms of utility, but are nevertheless invaluable to the degree or to the extent that they are interpretations of natural law in terms of human understanding. It is these inspired few to whom the phenomena of nature manifest themselves in the light of Walt Whitman's epigram, "The mountain and the pismire are equally wonderful"—and verily they have their reward.

The remaining groups reflect the need for immediate utilitarian results that can be at once applied in practice, and it is in this field that it is evident that co-ordination and sympathetic co-operation would be most helpful in achieving the ends sought.

A vast amount of scientific research work is being done in the industrial field of dentistry which has its direct bearing upon the accuracy, the precision and rationalism with which dental therapy is applied in professional practice. Many of the so-called professional problems which are the subject of professional research and study are already worked out or are engaging the attention of workers in the industrial field, and if the sentimental, artificial barriers between the professional group and the scientific experts who are catering to their requirements were eliminated the way would be cleared for a more rapid scientific advancement in dental practice and the present wastefulness in time, energy and brains involved in dental scientific research would be eliminated.—Editorial in *Dental Cosmos* for June, 1920.

